# The Production of Methane, Hydrogen, and Organic Compounds in Ultramafic-Hosted Hydrothermal Vents of the Mid-Atlantic Ridge

**DOI:** 10.1089/ast.2014.1198

**Published:** 2015-05-01

**Authors:** C. Konn, J.L. Charlou, N.G. Holm, O. Mousis

**Affiliations:** ^1^ Ifremer, Unité Géosciences Marine, Laboratoire de Géochime et Métallogénie, F-29280 Plouzané, France.; ^2^ Department of Geological Sciences, Stockholm University, SE-10691 Stockholm, Sweden.; ^3^ Aix Marseille Université, CNRS, LAM (Laboratoire d'Astrophysique de Marseille) UMR 7326, Marseille, France.

## Abstract

Both hydrogen and methane are consistently discharged in large quantities in hydrothermal fluids issued from ultramafic-hosted hydrothermal fields discovered along the Mid-Atlantic Ridge. Considering the vast number of these fields discovered or inferred, hydrothermal fluxes represent a significant input of H_2_ and CH_4_ to the ocean. Although there are lines of evidence of their abiogenic formation from stable C and H isotope results, laboratory experiments, and thermodynamic data, neither their origin nor the reaction pathways generating these gases have been fully constrained yet. Organic compounds detected in the fluids may also be derived from abiotic reactions. Although thermodynamics are favorable and extensive experimental work has been done on Fischer-Tropsch-type reactions, for instance, nothing is clear yet about their origin and formation mechanism from actual data. Since chemolithotrophic microbial communities commonly colonize hydrothermal vents, biogenic and thermogenic processes are likely to contribute to the production of H_2_, CH_4_, and other organic compounds. There seems to be a consensus toward a mixed origin (both sources and processes) that is consistent with the ambiguous nature of the isotopic data. But the question that remains is, to what proportions? More systematic experiments as well as integrated geochemical approaches are needed to disentangle hydrothermal geochemistry. This understanding is of prime importance considering the implications of hydrothermal H_2_, CH_4_, and organic compounds for the ocean global budget, global cycles, and the origin of life. Key Words: Hydrogen—Methane—Organics—MAR—Abiotic synthesis—Serpentinization—Ultramafic-hosted hydrothermal vents. Astrobiology 15, 381–399.

## 1. Introduction

**FIG. 1.**
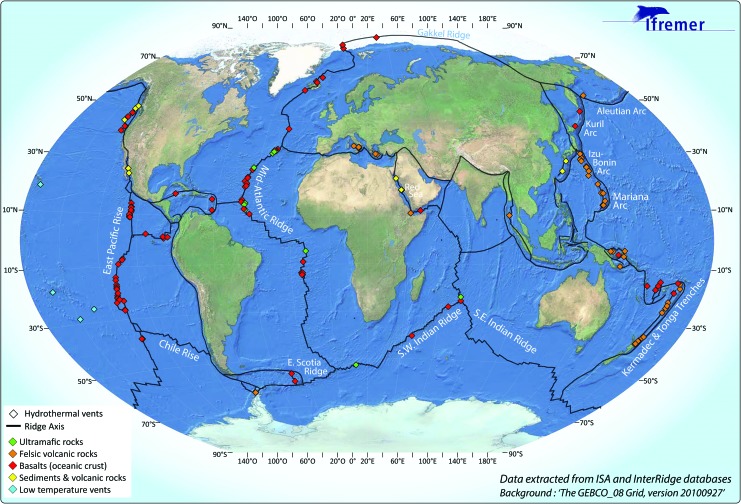
The MOR system showing the presently known and sampled hydrothermal sites. (Color graphics available at www.liebertonline.com/ast)

Hydrothermal circulation is a common process along oceanic spreading centers. The visible expression of this subsurface circulation is hydrothermal fields at the seafloor that are the foci of submarine oases of life. More than 500 sites have been located or inferred along the Mid-Ocean Ridge (MOR) system, including fast-, ultrafast-, slow-, and ultraslow-spreading ridges, as well as in back-arc basins, and many more are to be discovered based on the assumption of about 1 field every 100 km of ridge. More than 250 fields have been confirmed active and studied during oceanographic cruises with submersibles and/or remotely operated vehicles (Fig. 1).

**FIG. 2.**
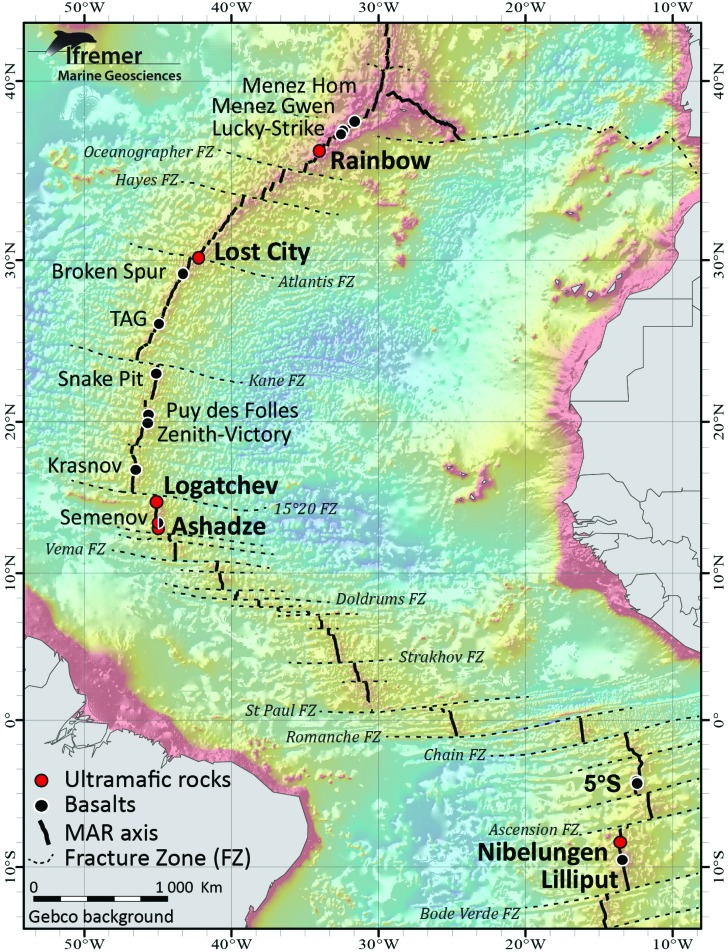
The MAR axis between 10°S and 45°N showing the known hydrothermal vent fields. Black circles represent basalt-hosted hydrothermal fields. Red circles represent ultramafic-hosted vent fields. (Color graphics available at www.liebertonline.com/ast)

Hydrothermal circulation occurs when seawater percolates downward through fractured ocean crust. The heated seawater is transformed into hydrothermal fluid through reaction with the host rock at temperatures that can exceed 400°C and exhaled forming thick smoke-like plumes of black metal sulfides often termed “black smokers.” During its transit through the oceanic crust, seawater composition is mainly modified by phase separation and water-rock interactions but is also influenced by (micro)biological processes and magmatic degassing. As a result, fluids become enriched in a variety of compounds and depleted in some others. In spite of their similar appearance, high-temperature hydrothermal vent fluids exhibit a wide range of temperatures and chemical compositions depending on subsurface reaction conditions and nature of the leached rocks (basalts, ultramafic rocks, felsic rocks). Notably, alteration of ultramafic rocks is associated with hydrogen release, which leads to high reducing conditions in these environments. In turn, it has been suggested that these reducing conditions would be favorable for the abiogenic production of methane and other organic molecules. One of the major implications for abiogenic synthesis is the origin of life. In this paper, we will discuss the origin of H_2_, CH_4_, and organic compounds in the deep sea and in ultramafic-hosted vents on the Mid-Atlantic Ridge (MAR) where most of these particular systems have been reported (Fig. 2). Particular attention is given to the use and helpfulness of stable isotopes in addressing the question of origin. We also present estimations of fluxes of hydrothermal H_2_ and CH_4_ entering the ocean and show that hydrothermal inputs are significant and should be considered in ocean global cycles studies. Finally, organic abiotic synthesis feasibility and implications for our understanding of the origin of life are the focus of the second part of the paper.

## 2. H_2_ and CH_4_ Origins in the Deep-Sea Environment

Molecular hydrogen is produced by biotic and abiotic processes, but its concentration in natural systems is usually very low due to the activity of hydrogen-consuming bacteria (Libert *et al.*, 2011; Petersen *et al.*, 2011). Heterotrophic bacteria produce hydrogen by fermentation of organic matter or by anaerobic oxidation of carbon monoxide (Silva *et al.*, 2000; Sokolova *et al.*, 2004; Pawar and van Niel, 2013). The evidence of a hydrogen-based subsurface microbial ecosystem was brought up by Takai *et al.* (2004a). More recently, the presence of communities capable of producing and oxidizing H_2_ has been reported at the Lost City vent field (Brazelton *et al.*, 2012). Hydrogen production by abiogenic processes includes deep crustal outgassing (Okuchi, 1997; Karato, 2006), crystallization of the basaltic magma (Christie *et al.*, 1986; Holloway and O'Day, 1999, 2000), and low-temperature reactions that occur in the shallow crust, and it is often related to active faulting (Wakita *et al.*, 1980; Sugisaki *et al.*, 1983; Ware *et al.*, 1985; Ito *et al.*, 1999). Active volcanic or seismic activities generate volatile compounds such as H_2_, CO, and H_2_S, although CO_2_ generally dominates, sometimes spectacularly (Sarda and Graham, 1990; Javoy and Pineau, 1991; Dixon *et al.*, 1995; Soule *et al.*, 2012). In addition, the radiolytic dissociation of water during the radioactive decay of natural U, Th, and K radionuclides in the host rock is another potential source of H_2_ (Lin *et al.*, 2005). Hydrogen may also be derived from small amounts of water included in minerals in the form of hydroxyls or peroxides (Freund *et al.*, 2002). Finally, and more to the focus of this paper, the production of H_2_ may be caused by the interaction of water at temperatures ranging from below 100°C up to 500°C with highly reduced iron-containing minerals occurring in ultramafic diapirs present in continental or submarine environments (Mevel, 2003). These so-called serpentinization reactions are likely to represent the dominant process for abiogenic formation of H_2_ in ultramafic-hosted hydrothermal systems and most efficiently at temperatures around 300–350°C (Klein *et al.*, 2013). Hydrogen-rich fluids issued from the serpentinization process are discussed in Section 3 and by Sleep *et al.* (2004).

Methane in deep-sea environments has many sources, which are discussed in detail in a review by Martin Schoell (1988). Most of the commercial methane is thermally derived from petroleum and has a biogenic origin (Rice and Claypool, 1981). This methane is often called thermogenic. Under pressure and low temperature, methane forms a thermodynamically stable association with water. These solid compounds are called methane clathrates and are typically found in permafrost or associated with rocks and mud in deep seafloor environments. Besides, microbial methane is produced by bacteria and archaea in sediments, subsurface and hydrothermal vents via CO_2_ reduction and/or fermentation (*e.g.*, Whiticar *et al.*, 1986; Takai *et al.*, 2004b; Amend and Teske, 2005; Roussel *et al.*, 2011). The observations made along the MAR together with the methane-rich fluids found in gabbroic inclusions from the South West Indian Ridge (SWIR) indicate that plutonic rocks represent a potentially immense reservoir for abiogenic methane (Kelley *et al.*, 1993; Kelley, 1996; Evans, 1996; Kelley and Früh-Green, 1999). A prevalent hypothesis for the abiotic production of significant amounts of CH_4_ in the deep sea at high pressure and temperature is via catalytic reduction of certain carbon oxides in the presence of H_2_. Field data associated with laboratory experiments (McCollom and Seewald, 2001) show that CH_4_, together with H_2_, is a major emission by-product of serpentinization. CH_4_ outgassing associated with intense H_2_ output has consistently been observed in ultramafic settings such as in peridotites of the Oman ophiolite (Neal and Stanger, 1983), in serpentinized rocks of the Zambales ophiolite, Philippines (Abrajano *et al.*, 1988), in serpentine seamount drilled during ODP Leg 125 in the Mariana Forearc (Haggerty, 1991), and along MORs (Charlou *et al.*, 1991, 1993a, 1996a, 2010; Charlou and Donval, 1993; Kelley, 1996; Früh-Green and Kelley, 1998). Alternatively, reasonable processes of abiogenic CH_4_ formation at high *T* and *P* include the reduction of bicarbonate to graphite and methane (Holloway, 1984; Berndt *et al.*, 1996), the thermal decomposition of siderite (McCollom, 2003), and clay mineral–catalyzed reactions (Williams *et al.*, 2005). Finally, low-temperature (<150°C) production of CH_4_ would be possible by hydration of olivine without H_2_ mediation and would be more common than previously thought (Miura *et al.*, 2011; Suda *et al.*, 2014). For a complete examination of potential abiotic CH_4_ sources on Earth, the reader is directed toward the review by Etiope and Sherwood Lollar (2013).

## 3. Abiotic H_2_ and CH_4_ Production in Ultramafic Hydrothermal Systems on the MAR

All the ultramafic-hosted, hot-temperature hydrothermal fields discovered along the MAR are characterized by strong enrichment of dissolved H_2_ and CH_4_, with end-member concentrations covering a range of 10–16 mmol/kg for H_2_ and 1.7–2.5 mmol/kg for CH_4_. While either H_2_ or CH_4_ may also be enriched in basaltic-hosted hydrothermal systems in unsedimented settings under certain conditions (Von Damm, 1995; Lilley *et al.*, 1993), only serpentinization of mantle rocks produces the characteristic coupled constant enrichment of both gases observed in ultramafic-hosted hydrothermal systems (Charlou *et al.*, 2002, 2010; Douville *et al.*, 2002).

Along the MAR, enhanced permeability at the intersections of the rift valley with the fracture zones favors seawater circulation and serpentinization of lower-crustal and upper-mantle ultramafic rocks. Close to transform-ridge intersections, structural settings enhance fluid circulation and wall-rock reactions (Bougault *et al.*, 1990, 1993; Gracia *et al.*, 2000), generating ultramafic rock exposures and methane outputs (Bougault *et al.*, 1993; Charlou *et al.*, 1996a, 1998). Faulting facilitates hydrothermal circulation through ultramafic outcrops, amplifies serpentinization reactions, and accelerates hydrogen and hydrocarbon degassing, as predicted by theoretical calculations (McCollom and Shock, 1998; Wetzel and Shock, 2000).

Serpentinization is an ongoing process at depth in the seafloor that leads to significant changes in topography, the occurrence of diapiric serpentinite bodies, focused microseismic activity as a result of continuous cracking, and significant heat generation (Fyfe, 1974; Allen and Seyfried, 2004). Chemically, serpentinization is the hydration of the olivine and orthopyroxene minerals that mainly constitute the upper mantle. Highly reducing conditions can be generated during serpentinization as a result of, on the one hand, the oxidation of Fe(II) in olivine, pyroxene, and pyrrhotite to Fe(III) in magnetite and serpentine, and on the other hand, the reduction of hydrogen from water to H_2_ (Allen and Seyfried, 2003; Klein *et al.*, 2009; McCollom and Bach, 2009). Hydrogen production by serpentinization proceeds most effectively in ultramafic rocks because the minerals that form in these silica-poor rocks during alteration tend to exclude Fe(II) from their metal sites and to partly oxidize and precipitate iron in magnetite (McCollom and Seewald, 2001, 2006, 2007) and serpentine (Andreani *et al.*, 2013; Evans *et al.*, 2013). One of the serpentinization equations is (Moody, 1976; Neal and Stanger, 1983; Mevel, 2003):

**Figure:**
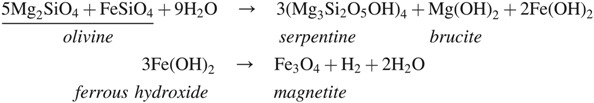


Phase equilibrium and mass balance calculations indicate that the production of H_2_-rich fluids during hydrothermal alteration is primarily controlled by the alteration of olivine and the formation of magnetite. It occurs when the lower crustal and shallow mantle sequences have cooled to temperatures below 400°C where serpentine and brucite are thermodynamically stable (Früh-Green and Kelley, 1998). Recent observations have revealed that this production is also widely controlled by the activity of aqueous silica in the interacting fluid, which is in turn controlled by the specific phase assemblages that develop during serpentinization (Frost and Beard, 2007; Ogasawara *et al.*, 2013). Depending on mineral assemblages, pressure, temperature, reducing power, and equilibrium conditions, the fluid may be supersaturated with respect to hydrogen. As the fluids ascend upward to lower pressures and temperatures, gas bubbles may be generated (Sleep *et al.*, 2004).

A consequence of this H_2_ production is the possible formation of abiogenic CH_4_. Among the different plausible reaction pathways, reduction of gaseous or dissolved carbon mono- and dioxides using catalysts such as Ni-Fe alloys and/or oxides has been by far the most studied and referred to (*e.g.*, Berndt *et al.*, 1996; Horita and Berndt, 1999; Chen and Bahnemann, 2000; Foustoukos and Seyfried, 2004; Taran *et al.*, 2010a). The latter is best described by Reactions 1 and 2:

\begin{align*}&( 1 ) \ \hbox{the Sabatier reaction}
\qquad\quad \ \ 4{ \rm H}_2 + { \rm CO}_2 \rightarrow{ \rm CH}_4 +
2{ \rm H}_2{ \rm O} \\& ( 2 ) \ \hbox{the Fischer  - Tropsch
reaction} \; 3{ \rm H}_2 + { \rm CO} \rightarrow { \rm CH}_4 + {
\rm H}_2 { \rm O}\end{align*}

Although CH_4_ and other hydrocarbons have been synthesized by Fischer-Tropsch-type (FTT) reaction in the gas phase from CO for more than 100 years (Anderson, 1984; Steynberg and Dry, 2004), these reactions can also proceed under aqueous hydrothermal conditions with CO_2_ as the carbon source (Berndt *et al.*, 1996; Horita and Berndt, 1999; Horita, 2001; McCollom and Seewald, 2001, 2006, 2007; Foustoukos and Seyfried, 2004; Seewald *et al.*, 2006). Experiments carried out at temperatures lower than 500°C combined with thermodynamically favorable conditions (Shock, 1990, 1994; Shock and Schulte, 1990, 1998; Amend and Shock, 1998) confirm that MORs' ultramafic hydrothermal systems may provide conditions that allow for abiogenic generation of CH_4_ from CO_2_ (Berndt *et al.*, 1996; Holm and Andersson, 1998; Holm and Charlou, 2001; Kelley *et al.*, 2002).

## 4. Fluxes of H_2_, CH_4_ along the MAR

It is now demonstrated that the concentrations of methane and hydrogen in hydrothermal vent fluids are very variable. High concentrations of these gases are found in ultramafic-hosted vent fields (Table 1), whereas relatively lower concentrations are found in basalt-hosted vent fluids. Exceptions are fluids from the Menez Gwen, Lucky Strike, and more recently discovered Piccard vent field usually referred to as basalt-hosted vents. Their concentrations of CH_4_ are similar to, and sometimes exceed, the ones observed in ultramafic-hosted vents. Lucky Strike has consistently vented high CH_4_ but low H_2_ fluids for almost three decades, which could be related to magmatic events (Von Damm *et al.*, 1998; Charlou *et al.*, 2000; Pester *et al.*, 2012; Crawford *et al.*, 2013). As for the Menez Gwen and Piccard vent fields, the occurrence of ultramafic rocks at depth or in the vicinity cannot be excluded (Stroup and Fox, 1981; Charlou *et al.*, 2002). However, if one discards this option, high production of CH_4_ and H_2_ in those basaltic environments could be due to the reaction of water with CO_2_ from direct magmatic degassing or leaching from CH_4_-rich inclusions in gabbroic rocks (Kelley *et al.*, 1993; Kelley, 1996), with respect to CH_4_, and to water cracking related to dike formation or to the alteration of troctolites, with respect to H_2_ (Elthon, 1987; Nakamura *et al.*, 2009).

**Table 1.** Gas End-Member Data in Ultramafic Fluids from the MAR

*Element*	*Lost City*^a^*30°07′N*	*Rainbow*^b^*36°14′N*	*Logatchev 1*^c^*14°45′N*	*Logatchev 2*^d^*14°45′N*	*Ashadze 1*^e^*12°58′N*	*Ashadze 2*^f^*12°59′N*
Best sample	EXO-D17-Ti4	EXO-D6-Ti4	SE-DV7-Ti3-L1	SE-DV7-Ti3-L2	SE-DV2-Ti2	SE-DV4-Ti3
*T* (°C) max	94	365	359	320	372	>296
pH	12.1	3	3.9	4.2	3.1	4.1
Total gas volume (NTP) (mL/kg)	211	813	525	527	687	776
H_2_ (m*M*)	7.8	12.9	12.5	11.1	19	26.5
CO_2_ (m*M*)	—	17	4.4	6.2	3.7	nd
CH_4_ (m*M*)	0.9	1.65	2.6	1.2	1.2	0.8
C_2_H_6_ (μ*M*)	0.67	0.83	0.77	0.19	0.17	5.7
C_3_H_8_ (μ*M*)	0.070	0.046	0.024	0.011	0.020	0.21
C_1_/C_2+_ (10^3^)	1.22	1.88	3.27	5.67	6.32	0.14

*Data sources:* ^a,b^Data from Exomar (2006) cruise of Ifremer. ^c,d,e,f^Data from Serpentine (2007) cruise of Ifremer.

nd: not detected. NTP: normal temperature and pressure.

Mid-Ocean Ridge fluxes of hydrogen and methane at oceanic spreading centers appear nonetheless to be mainly supplied by serpentinization of ultramafic rocks. The total hydrogen and methane produced by serpentinization is estimated to be 133×10^9^ mol/yr and 14×10^9^ mol/yr, respectively (Keir, 2010), representing about 70% of the total ocean ridges and rises flux of these gases. These fluxes are slightly lower than those calculated by Cannat *et al.* (2010) according to the rate of mantle rock exhumation and the stoichiometry of olivine hydration (167×10^9^ mol/yr and 25×10^9^ mol/yr for H_2_ and CH_4_, respectively). Emmanuel and Ague (2007) obtained even higher values for the flux of CH_4_ generated by serpentinized lithosphere based on estimates of the rate of seafloor spreading and the degree of serpentinization within the oceanic crust. They calculated it to be 84×10^9^ mol/yr. In comparison, volcanic and geothermal sources are estimated to contribute for ∼6.2×10^9^ mol/yr and ∼56×10^9^ mol/yr, respectively. Based on Rainbow H_2_/^3^He and ^3^He/heat ratios (^3^He/heat=9.3×10^8^ mol J^−1^ in Jean-Baptiste *et al.*, 2004), global H_2_ and CH_4_ fluxes for slow spreading ridges have been calculated to be 89×10^9^ mol/yr and 9×10^9^ mol/yr, respectively (Charlou *et al.*, 2010). All these results are within the uncertainty of plus/minus a factor of 2 inherent to these estimates, but they indicate the necessity of taking hydrothermal gas inputs into account in global cycles studies.

## 5. Origin of CH_4_ in the Fluids of Ultramafic-Hosted Vents on the MAR from H and C Stable Isotopes

**FIG. 3.**
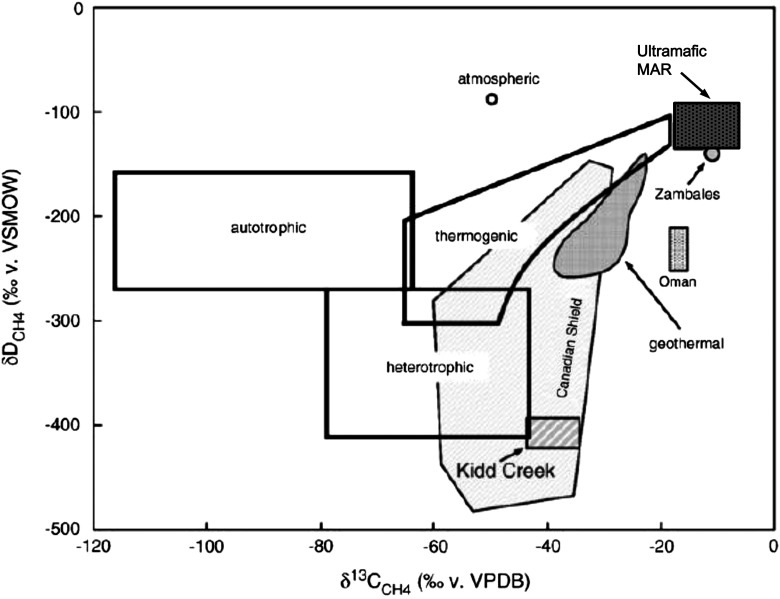
Modified after Bradley and Summons (2010). Ranges of δ^13^C and δD detected in methane produced by a variety of sources. “Autotrophic” and “heterotrophic” is microbial methane. “Thermogenic” refers to cracking of biologically derived oils, while “geothermal” refers to cracking of high-molecular-weight organic compounds. The remaining are values observed at several locations where abiotic methane formation has been suggested: Canadian Shield gases (including Kidd Creek), the Oman ophiolite, Zambales ophiolite. The dark surface represents the range of values (from Table 2) measured for methane in fluids from the Rainbow, Lost City, Logatchev 1 and 2, Ashadze 1 and 2 ultramafic-hosted vent fields.

The possible contribution to the methane budget in fluids from ultramafic-hosted vents may be multiple, as mentioned before and discussed in a review by McCollom (2008). Isotopic considerations appear compulsory to unravel the origin of CH_4_. Several authors have suggested that abiogenic methane is, or was, produced in various geological settings such as crystalline rocks, ophiolites, gas seepages, fumarolic discharges, and ultramafic-hosted hydrothermal systems on land, based on carbon and hydrogen isotope measurements (Sherwood Lollar *et al.*, 1993, 2006; Fiebig *et al.*, 2004, 2007, 2009; Hosgormez *et al.*, 2008; Taran *et al.*, 2010b; Etiope *et al.*, 2011; Suda *et al.*, 2014). The consensus is that it is formed via the Sabatier and FTT pathways (Reactions 1 and 2). In ultramafic-hosted fluids collected up to now along the MAR, the δ^13^C_(CH4)_ value is found to be −11.9 ‰ at Lost City, −17.8 ‰ at Rainbow, −10.2 ‰ at Logatchev 1, −6.1 ‰ at Logatchev 2, −12.3 ‰ at Ashadze 1, and −8.7 ‰ at Ashadze 2 (Table 2). These results combined with the δD_(CH4)_ fall into a range that is neither thermogenic nor biogenic as previously found in other hydrothermal fluids and shown in Fig. 3 (Schoell, 1988; Welhan, 1988; Charlou *et al.*, 1993b, 1996b, 2000). The same trend is observed when considering δ^13^C_(CH4)_ versus the CH_4_-to-higher-hydrocarbons ratio (C_1_/C_2+_) (Table 2). The results for Rainbow, Lost City, Logatchev 1 and 2, and Ashadze 1 are consistent with typical values for unsedimented MOR systems, whereas Ashadze 2 has a lower C_1_/C_2+_ ratio and is closer to the Zambales ophiolite values (McCollom, 2008). Only an abiogenic contribution may account for these observations. The variability in δ^13^C_(CH4)_ observed here and also reported in the literature is probably due to fractionation at different *T* and *P* conditions. Nevertheless, there is a clear abiogenic contribution to the CH_4_ budget in hydrothermal fluids issued from ultramafic environments, which is supported by experiments (Horita and Berndt, 1999; Horita, 2001; Lazar *et al.*, 2012; Cao *et al.*, 2014).

**Table 2.** Carbon and Hydrogen Isotope Data from Ultramafic Fluids from the MAR

*Hydrothermal field*	*Sample*	T *(°C)*	δ^13^*C-CH_4_*	δ*D-CH_4_*	δ^13^*C-C_2_H_6_*	δ*D-C_2_H_6_*	δ^13^*C-C_3_H_8_*	δ^13^*C-C_4_H_10_*	δ^13^*C-CO_2_*	δ*D-H_2_*
Lost City^a^	3876-GT7	90	−11.0	−127	−13.5	—	−14.5	−14.6	−8 to +3^*^	−609^**^
Lost City^b^	EXO-D17-Ti4	93	−11.9	−130	−13.7	−148	−14.0	−12.6	—	−618
Rainbow^b^	EXO-D7-Ti1	343	−17.7	−105	−13.7	—	−13.0	−13.2	−2.3	−356
	EXO-D9-Ti4	324	−17.8	−107	−13.4	—	−13.0	—	—	−379
Logatchev 1^c^	SE-D6-Ti3	346	−10.2	−104	−5	—	−18.0	—	4.1	−350
	SE-D7-Ti3L1	325	−10.3	−104	−13	—	−8	—	7.4	−360
Logatchev 2^c^	SE-D7-Ti3L2	308	−6.1	−93	−9	—	−11.0	−12.2	9.5	−231
Ashadze 1^c^	SE-D2-Ti3	353	−12.3	−104	−6	—	−20	−19.2	2.1	−333
	SE-D3-Ti3	353	−14.1	−101	−10	—	−18	—	4.6	−343
Ashadze 2^c^	SE-D4-Ti3	>296	−8.7	−107	−2.0	—	8.3	10.0	0.2	−270

*Data sources:* ^a^Proskurowski *et al.* (2008); ^b^Exomar cruise data (2005); ^c^Serpentine cruise data (2007); ^*^Kelley *et al.* (2005); ^**^Proskurowski *et al.* (2006).

All isotope values are in ‰ units; δ^13^C is reported as VPDB and δD as VSMOW.

δ^13^C-CH_4_ measurement uncertainty is ±0.2‰. δ^13^C-C_2_H_6_ and C_3_H_8_ measurement uncertainty is 0.3‰.

δ^13^D-CH_4_ measurement uncertainty is ±1‰. δ^13^D-C_2_H_6_ measurement uncertainty is±3.5‰.

The number of ultramafic-hosted sites discovered along the MAR associated with the detection of numerous CH_4_-rich plumes associated with mantle rock alteration indicates that ultramafic-hosted sites are more widespread than previously thought (Charlou and Donval 1993; Charlou *et al.*, 1993a; Keir *et al.*, 2005). This points out their significant contribution to the global abiogenic methane flux along the MAR (Charlou *et al.*, 1991, 1996a, 1996b, 1998; Bougault *et al.*, 1993). This is also supported by Keir *et al.* (2005), who estimated the amount of methane escaping from the MAR to be equivalent to about 0.06 nmol per liter of mid-depth water and per year. Extrapolated to the whole Atlantic, this comes to about 1×10^9^ mol/yr. The methane production required to support this escape rate (>3.6×10^9^ mol/yr) is significantly greater than the maximum input by basalt degassing (<0.6×10^9^ mol/yr)—evidence that serpentinization of ultramafic rocks generates most of the methane and is the main active process producing isotopically heavy methane along the MAR.

## 6. Organic Geochemistry—Pathways

Organic compounds have been found in hydrothermal fluids and investigated since the 2000s. Semivolatile ones (>6 carbon chain length) such as aliphatic hydrocarbons, mono- and polyaromatic hydrocarbons, and carboxylic acids have been reported on the MAR (Holm and Charlou, 2001; Konn *et al.*, 2009; McCollom *et al.*, 2015). Formate (36–158 μ*M*) and acetate (1–35 μ*M*) have been observed by Lang *et al.* (2010) in fluids from the Lost City hydrothermal field. Reeves *et al.* (2014) measured concentrations of 10^−9^ to 10^−6^ *M* of methanethiol in fluids from hydrothermal vents in various geological settings including the MAR. Amino acids were found in fluids from various sites of the MAR. The total dissolved free amino acid concentrations were up to 377 n*M* versus <50 n*M* for deep seawater (Sumoondur *et al.*, 2006; Klevenz *et al.*, 2010). Organic geochemistry of hydrothermal fluids is a brand new field that raises a lot of questions, in particular regarding the origin of organic compounds and their potential importance for the origin of life on Earth. The processes that control the organic composition of the fluids are not yet fully constrained or understood. Both the sources of the building units composing organic molecules (C, H, O, and N) and possible reaction pathways leading to organic compounds are multiple; therefore, determining the origin of organic compounds and understanding their formation appears a real challenge. On the one hand, rocks, minerals, and seawater are potential C, H, O, and N sources; in a simplified view, CO_2_ and carbonates are sources of C, H_2_ and water sources of H, N_2_ and NH_3_ sources of N, water and oxygen-bearing minerals sources of O. On the other hand, microorganisms that inhabit hydrothermal vents and the subsurface may provide C, H, O, and N by two mechanisms: (i) direct production of simple molecules (*e.g.*, CH_4_, H_2_, acetate, CO_2_), (ii) thermal degradation of the microorganisms themselves if exposed to high-temperature fluids. In addition, macroorganisms may also undergo thermal degradation after death and sedimentation. Whether C, H, O, N are derived from gas, minerals, and seawater or from organisms, they will be referred to as abiogenic or biogenic, respectively. Multiple processes possibly leading to the formation of organic molecules from those C, H, O, N sources in hydrothermal environments include abiogenic processes that represent any purely chemical reactions; biogenic processes that encompass all reactions driven by microorganisms; and thermogenic processes that refer to both thermal degradation of large organic molecules (*e.g.*, proteins, lipids, DNA) to smaller and simpler ones as well as rearrangement of compounds under high temperature and pressure conditions such as condensation, cleavage, cyclization, hydrolysis, oxidation, hydrogenation, and hydroformylation (Rushdi and Simoneit, 2004, 2006; Loison *et al.*, 2010).

### 6.1. Abiogenic processes

Abiotic synthesis will only occur if thermodynamics are favorable. This has been shown to be the case for a wide range of organic compounds under conditions found at modern subseafloor hydrothermal systems (*e.g.*, Shock, 1990; McCollom and Seewald, 2001; Lemke, 2013). The hypothesis is that organic compounds would occur in metastable equilibrium due to kinetic barriers that would prevent the inherently sluggish stable equilibria CO_2_/CH_4_ and N_2_/NH_3_ to be reached in hydrothermal solutions. We refer to the work of Shock (1990, 1992) and Konn *et al.* (2009) for a more in-depth discussion. Reaction pathways are not well constrained, although mechanisms have been proposed for hydrocarbons and amino acids.

#### 6.1.1. Hydrocarbons—FTT reactions

Abiogenic origin of hydrocarbons was first brought up by Mendeleev in 1877 and has been highly debated since 1940 (*e.g.*, Mendeleev, 1877; Kudryavtsev, 1951; Hedberg, 1969; Szatmari, 1989; Gold, 1993; Kutcherov and Krayushkin, 2010). The two recent reviews published in 2013 by Sephton and Hazen, on the one hand, and by Etiope and Sherwood Lollar, on the other hand, are evidence that this debate is still fueled. Today, technological advances have helped clarify the various controversial theories that were elaborated on. A fairly recent review by Glasby (2006) highlights the lack of strong evidence to support the abiogenic petroleum theories and rules out the abiogenic production of oil in commercial quantities. Notably, this does not exclude the possibility of a minor abiotic contribution (Jenden, 1993; Sherwood Lollar *et al.*, 2002). Apps and van de Kamp (1993) concluded that, even though commercial hydrocarbon deposits appear to be exclusively biogenic in origin, this may be at the exception of deposits associated with serpentinization. In 1964, a mixed origin of hydrocarbons was implied (Sylvester-Bradley). With the advent of modern analytical tools, the co-occurrence of both biogenic and abiogenic signatures in most hydrocarbon fields has been confidently revealed (Mello and Moldowan, 2005; Scalera, 2011). In that respect, Scalera (2011) developed a possible new harmonic scenario of hydrocarbon formation combining both thermogenic and abiogenic processes. It seems that scientists are moving toward a consensus that hydrocarbons may be produced by different pathways on Earth, but the question that remains is what the contribution of each process is both globally and in specific geological contexts. In ultramafic-hosted hydrothermal systems, several processes are capable of generating reduced carbon species (Seewald *et al.*, 2006). These include FTT reactions, methane polymerization, carbonate decomposition, organosulfur pathways, and clay-catalyzed reactions. They are presented in detail in a review by McCollom (2013b). Among them, FTT reactions have been addressed by extensive experimental work under hydrothermal conditions in the past decades. FTT processes are considered prime candidates to account for the generation of abiogenic hydrocarbons in ultramafic-hosted hydrothermal systems (Holm and Charlou, 2001; Konn *et al.*, 2009).

The Fischer-Tropsch reaction (3) was a common industrial process used to produce hydrocarbons from CO and H_2_ (Fischer and Tropsch, 1923). It was invented by two German scientists, Franz Fischer and Hans Tropsch, in the 1920s and was largely developed during World War II to generate substitute fuels. The original process takes place in the gas phase at high pressure and temperature according to the following mass balance equation:

\begin{align*}( 2n + 1 )\ { \rm H}_2 + n{ \rm CO} \rightarrow {
\rm C}_n {\rm H}_{2n + 2} + n { \rm H}_2 { \rm O}
\tag{3}\end{align*}

Numerous laboratory experiments conducted under hydrothermal conditions have demonstrated the feasibility of the abiogenic production of hydrocarbons (*e.g.*, McCollom and Simoneit, 1999; McCollom *et al.*, 1999; Rushdi and Simoneit, 2001; Foustoukos and Seyfried 2004; McCollom and Seewald, 2006). Thermodynamic calculations have shown that saturated hydrocarbons can be abiotically produced via FTT reactions under hydrothermal conditions from dissolved CO_2_ (Shock, 1990, 1992). The reactions involved in FTT reduction of aqueous CO_2_ can be expressed as follows:

\begin{align*}{ \rm CO}_2 { \rm ( aq ) } + [ 2 + ( m / 2n ) ] {
\rm H}_2 \rightarrow ( 1 / n ) { \rm C}_n { \rm H}_n + 2{ \rm H}_2
{ \rm O} \tag{4}\end{align*}

The distribution of the observed hydrocarbons makes it quite clear that they are the product of FTT reactions, but isotopically labeled experiments have shown that CO is a much more effective precursor than CO_2_ (McCollom and Seewald, 2001; McCollom *et al.*, 2010). It is still unclear whether the reactions occur in the gas phase or in the water phase, which could favor one or the other oxidized form of C to react preferentially. The exact pathways along which these hydrocarbons are formed are not fully constrained yet, either, and the actual occurrence of FTT processes at deep-sea hydrothermal conditions (where CO_2_, CO, H_2_ are dissolved) is still uncertain. Although every single experiment has been a great step forward, their results are in most cases not intercomparable because of crucial differences in the experimental conditions. Parameters such as *P*, *T*, redox state, presence/absence of a catalyst, and carbon source significantly impact the resulting products and most likely the involved processes. Unraveling the reaction pathways is a real challenge considering this unfortunate inconsistency in the overall set of published experiments. We urge the reader to refer to the work of McCollom and Seewald (2007) and McCollom (2013b) for a detailed review.

#### 6.1.2. Amino acids—Strecker synthesis

The abiotic synthesis of amino acids is of particular interest in the origin of life question, as they represent the fundamental building blocks of proteins that are required for the development of living organisms. The amino acid synthesis has been generally proposed to occur via a Strecker-type mechanism under hydrothermal conditions (*e.g.*, Hennet *et al.*, 1992; Schulte and Shock, 1995; Islam *et al.*, 2001; Aubrey *et al.*, 2009). The original Strecker amino acid synthesis, devised by Adolph Strecker in 1850, is a series of chemical reactions that synthesize an amino acid from an aldehyde (or ketone) according to Reaction 5 (Strecker, 1850):

**Figure:**
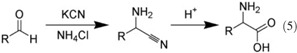

The Strecker synthesis has been shown to be thermodynamically favorable over all ranges of temperatures appropriate for a hydrothermal system at 300 bar (Brandes *et al.*, 1998). Nevertheless, for this reaction pathway to proceed in hydrothermal systems the formation of the required reactants (HCN and aldehyde or ketone) by reduction of inorganic carbon (CO or CO_2_) and nitrogen (N_2_) must first occur. In that respect, highly reducing conditions encountered in ultramafic-hosted hydrothermal environments are very favorable. Formation of HCN from N_2_ and CO_2_ in the presence of H_2_, possibly with CH_4_ as an intermediate, is both thermodynamically and experimentally strongly supported (Shock, 1992; Holm and Neubeck, 2009). Experimental works have shown that amino acids are likely formed under hydrothermal conditions and more favorably under high hydrogen concentrations (Hennet *et al.*, 1992; Islam *et al.*, 2001; Huber and Wächtershäuser, 2006; Simoneit *et al.*, 2007). Unless they are protected by mineral surfaces or undergo polymerization or cyclization (Ito *et al.*, 2006; Cox and Seward, 2007), amino acids are likely to be destroyed by deamination, decarboxylation, and dehydration at temperatures above 240–260°C and even at ∼170°C in the presence of certain mineral assemblages (Bada *et al.*, 1995; Faisal *et al.*, 2007; McCollom, 2013a). The possible occurrence and persistence of amino acids in hydrothermal fluids thus depends at least on redox conditions, mineral assemblages, temperature, and pressure.

To date, there is no evidence that abiotic amino acid synthesis occurs in natural environments. Undeniably, amino acids have been found in fluids of hydrothermal systems in various geological settings, including ultramafic-hosted hydrothermal vents, but unanimously the authors reporting these amino acids have concluded that they are most likely derived from microorganisms living on the surface of the chimney (Horiuchi *et al.*, 2004; Takano *et al.*, 2004; Sumoondur *et al.*, 2006; Klevenz *et al.*, 2010; Lang *et al.*, 2013; Fuchida *et al.*, 2014). To complement the set of data on amino acids, Table 3 (from Konn *et al.*, 2015) gives some preliminary results of fluids from other vents on the MAR. Consistent with the above-cited works, only a portion of the entire set of amino acids was detected. It is probably due to different limits of detection, degradation rates, as well as different abilities to polymerize and to adsorb on mineral surfaces (Gupta *et al.*, 1983; Henrichs and Sugai, 1993).

**Table 3.** Hydrothermal Fluid Samples Main Features

									*C in hydrothermal fluid (ppt)*									
*Sample name*	*Site*	*Description*	*Depth (m)*	T*(°C)*	*pH*	*Cl^-^m*M	*Fluid %*	*Pre-C fold*	*Glu*	*Ala*	*Met*	*Trp*	*Pro*	*Gly*	*Lys*	*Tyr*	*Phe*	*Leu*
MAD-D2-Ti2D	seawater	reference	2291	2	7.84	550	nm	30	nd	344	nd	nd	nd	nd	nd	nd	nd	x
MAD-D3-Ti3G	Rainbow	black smoker	2307	350	3.23	761	97	25	nd	nd	nd	x	nd	nd	nd	nd	nd	nd
MAD-D6-Ti3D^*^	Rainbow	black smoker	2265	253	4.72	638	40	50	nd	69	x	nd	nd	nd	nd	nd	x	x
MAD-D6-Ti2G	Rainbow	black smoker	2265	353	3.41	711	73	40	nd	nd	nd	nd	nd	nd	nd	nd	x	x
MAD-D8-Ti1D	Rainbow	black smoker	2305	350	3.36	703	71	25	nd	nd	nd	nd	x	nd	nd	nd	x	x
MAD-D8-Ti3D	Rainbow	diffuser	2297	52.5	6.34	560	7	40	nd	41	nd	nd	nd	nd	nd	nd	x	x
SE-D2-Ti2	Ashadze 1	black smoker	4088	353	3.95	595	76	25	nd	nd	nd	x	nd	nd	nd	nd	x	54.2
SE-D2-Ti3	Ashadze 1	black smoker	4088	353	3.89	601	81	33	nd	nd	nd	x	nd	nd	nd	nd	x	14.9
SE-D3-Ti4^*^	Ashadze 1	black smoker	4088	355	4.13	604	81	25	nd	nd	nd	x	nd	nd	nd	nd	x	x
SE-D4-Ti3	Ashadze 2	black smoker	>3263	—	6.17	452	36	50	nd	nd	nd	nd	nd	nd	nd	nd	x	x
SE-D6-Ti1	Logatchev 1	black smoker	3021	346	4.97	517	71	50	nd	nd	nd	nd	nd	nd	nd	nd	x	x
SE-D7-Ti1-L2	Logatchev 2	black smoker	2700	308	4.44	171	93	50	nd	nd	nd	nd	nd	nd	nd	nd	x	x

As seawater mixing occurred in some samples, the % of pure fluid is given in the Fluid column. The Pre-C column gives how much the samples were concentrated before analyses (in fold). Concentrations are given in ppt, *i.e.*, in ng L^−1^, and refer to the concentration in the natural hydrothermal fluid originally, before preconcentration.

^*^ Spiked samples with 50 μL of the 1000-fold diluted standard solution.

nd: not detected. nm: not measured.

## 6.2. Biogenic processes

Chemolithotrophic microbial communities commonly colonize hydrothermal vents and may represent analogues for life on early Earth and other planets. Chemolithotrophic organisms, by definition, utilize only inorganic and/or abiotic simple molecules for their carbon and energy sources so that they do not rely on other living organisms to feed, develop, and multiply (Lang *et al.*, 2012). To date, the maximum temperature for some of such organisms to grow is 122°C (Takai *et al.*, 2008).

In our case, the archaea methanogens, which are one of the most common microorganism groups found at hydrothermal vents, are of particular interest, as they synthesize CH_4_ from CO_2_ and H_2_ (Schoell, 1988; Takai *et al.*, 2004b; Brazelton *et al.*, 2011; Nishizawa *et al.*, 2014). The consumption of methane leading to the production of CO_2_ by methanotrophic bacteria occurs to a lesser extent because methanotrophs are less abundant in hydrothermal environments. Also, from CO_2_ and H_2_, acetogenic bacteria are able to generate acetate which can, in turn, be used as substrate by heterotrophic methanogens (*e.g.*, Chapelle and Bradley, 1996). As mentioned earlier, the majority of amino acids detected in hydrothermal fluids is thought to be microbially derived partly because the autotrophic synthesis of several amino acids from CO_2_(aq), NH_4_^+^, and H_2_ is thermodynamically favorable at hydrothermal conditions (Amend and Shock, 1998).

## 6.3. Thermogenic processes

Typically, thermogenic processes occur in sedimentary basins and are associated with maturation of petroleum, which is defined as the oil and gas generated during thermolysis from the former (*e.g.*, Demaison and Murris, 1984; Tissot and Welte, 1984). Hydrothermal systems definitely meet the condition of high temperature required for thermal degradation (see reviews in McCollom and Seewald, 2007; McCollom, 2008). Organic matter is present in the form of macroorganisms and microorganisms that thrive both around the chimneys and in the subsurface. Macroorganisms from the surface ocean will inevitably die and fall to the seafloor. Degradation products may be taken up by seawater and penetrate the crust in the recharge zone of hydrothermal systems and thus undergo thermogenesis deeper in the crust (Brault *et al.*, 1988). Similarly, microbial organisms growing in the subsurface may be either flushed by a cold fluid and carried away to a place where temperature would be high enough to degrade the very durable lipids that form the membranes of the bacteria and archaea, or burned off as a very hot fluid would encounter these communities (Reeves *et al.*, 2014).

## 6.4. Biogenic versus abiogenic—the use of carbon stable isotopes

Organic compounds in hydrothermal systems are likely to result from co-occurring abiogenic, biogenic, and thermogenic processes using both biogenic and abiogenic C, H, O, and N and eventually leading to extensive mixing of biogenic and abiogenic C, H, O, and N elements within organic molecules. For example, biogenic methane, carbon dioxide, and acetate could well then be involved in abiotic processes, such as the previously described FTT and Strecker reactions. A more extensive discussion can be found in the work of McCollom (2008). This raises two issues: (i) How do we discriminate? (ii) What do we call those resulting organic compounds? They are neither biogenic nor abiogenic nor thermogenic. Table 4 is an attempt to illustrate this dilemma. We dare to suggest that, as long as terminology has not been agreed upon, distinction might be made between sources and processes. In the above-mentioned example, compounds might be called biogenic with respect to their source and abiogenic with respect to the process they were generated along. Our ability to classify organic compounds with more than two carbon atoms into the biogenic or abiogenic category might be very challenging (Horita, 2005). Even for the simplest organic molecule that is methane, the sole use of isotopic composition is sometimes insufficient, and complementary techniques have been used to determine its origin (*e.g.*, Bradley and Summons, 2010). Elsewhere, Etiope and Sherwood Lollar (2013) described the importance of integrated geochemical techniques to confirm the occurrence of abiogenic methane. It has been generally presumed that thermogenic, biogenic, and abiogenic hydrocarbons should differ in their carbon isotopic composition. Typical reference values of δ^13^C_(CH4)_ are −70‰ to −60‰ for a biological production, −60‰ to −40‰ for a thermogenic origin, −30‰ to −20‰ for geothermal hydrocarbons, and −20‰ to −5‰ for MORs (Schoell, 1988; Bradley and Summons, 2010), but this division is being debated (*e.g.*, Sherwood Lollar and McCollom, 2006; Ueno *et al.*, 2006a, 2006b). As for hydrocarbon gases (C_1_–C_4_), it has been suggested that a slight decrease in δ^13^C with increasing carbon number could be an indication of an abiotic catalytic formation, while a thermogenic origin has always shown a strongly positive correlation (Des Marais *et al.*, 1981; Sherwood Lollar *et al.*, 2002; Pan *et al.*, 2006). This isotope reversal trend has been attributed to kinetic isotope fractionation effects during surface-catalyzed polymerization reactions of methylene units (*e.g.*, Schoell, 1983; Jenden, 1993; Fu *et al.*, 2011). As the trend is weak to almost flat, it was even suggested that no fractionation occurs during polymerization. However, hydrocarbon gases produced experimentally via abiogenic reactions do not consistently produce inverse or flat trends, and results are rather heterogeneous (*e.g.*, McCollom and Seewald, 2006; Fu *et al.*, 2007; Taran *et al.*, 2007, 2010a; McCollom *et al.*, 2010). Experiments reported in the literature to date were carried out under various physical and chemical conditions. This strongly indicates that carbon isotope fractionation of hydrocarbons is controlled by their formation processes and kinetics, which in turn may differ according to temperature, pressure, and redox conditions (McCollom and Seewald, 2006; Fu *et al.*, 2011). Whether the experiments were conducted in a gas and/or water phase and in a closed or flow-through reactor are other possible influencing factors. This is discussed more in depth elsewhere (McCollom, 2013b). In addition, several thermogenic gases do show reversals of the kind attributable to abiotic reactions (*e.g.*, Burruss and Laughrey, 2010). The reverse or flat trend has generally been observed for hydrocarbon gases in ultramafic-hosted hydrothermal systems, but no clear evidence of their abiogenic origin has been brought forth (Proskurowski *et al.*, 2008; Charlou *et al.*, 2010). Clearly, it will be even more difficult to determine the origin of longer n-alkanes and other organic compounds detected in fluids from ultramafic-hosted vents.

**Table 4.** An Attempt to Highlight the Fact That Terminology Is Missing for Organic Compounds Resulting from Mixed Processes and Carbon Source

Source	Abiogenic	Biogenic
Processes	*(ex: mantle CO_2_)*	*(microbial production and organic matter degradation)*
Abiogenic *(ex: FTT)*	**Abiogenic**	**?**
Biogenic *(ex: methanogens)*	**?**	**Biogenic**
Thermogenic *(ex: cracking)*	**?**	**Thermogenic**

For example, a molecule resulting from FTT reaction using mantle CO_2_ will be called abiogenic. If the same process uses CO_2_ from respiration (although we do not know if this kind of reaction can actually occur, so we beg the reader to take this as an illustration), we currently do not know what to call the resulting product, which would be biogenic with respect to the source and abiogenic with respect to the process. Boxes left with a ? point out the word missing.

**FIG. 4.**
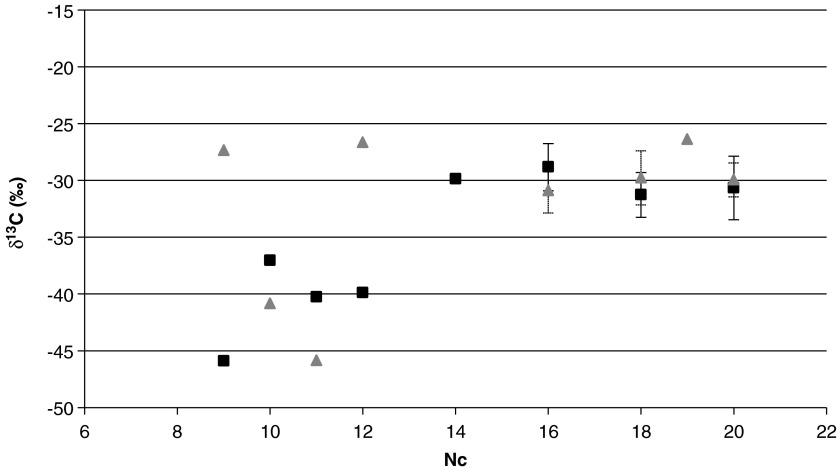
Carbon stable isotope ratios versus carbon number (Nc) for the n-alkane series detected in fluids from the Lost City field (triangles) and the Rainbow field (squares). C_9_–C_14_ (Konn *et al.*, 2009) and C_16_–C_20_ (Konn *et al.*, unpublished results). Analytical and sample collection methods are described by Konn *et al.* (2009).

Semivolatile organic compounds (>6 C atoms chain length) have rarely been reported as products of FTT reaction experiments probably due to their low concentration (*e.g.*, Taran *et al.*, 2007, 2010a). And, as far as the authors know, there are only two examples of an FTT experiment in which δ^13^C values of the heavy products (>C12) have been measured (McCollom and Seewald, 2006; McCollom *et al.*, 2010). These results indicate that different δ^13^C trends may be expected depending on the carbon source. Another influential parameter may be pressure (Fu *et al.*, 2011). Carbon isotopic ratios for the n-alkane series C_9_–C_20_ seem to show a different pattern (Fig. 4) from the one produced in McCollom's experiments (2010). Despite new experimental work and field data, the conclusion on the origin of hydrocarbons from various sources and/or processes drawn by Konn *et al.* (2009) remains.

Whereas Lang and coworkers (2010) were able to conclude with some confidence on the origin of formate (abiogenic) and acetate (microbially derived) using stable C isotopes, the origin of heavier fatty acids cannot be unraveled by using their individual δ^13^C value. Konn *et al.* (2009) showed that fatty acids were slightly enriched in ^13^C compared to the alkanes of the same chain length and inferred that this fractionation could possibly be created by biological processes.

It was generally admitted that amino acids were of biogenic origin in hydrothermal systems. Based on their carbon isotope measurements, Lang *et al.* (2013) were able to demonstrate that the amino acids in the hydrothermal fluids at Lost City were derived from chemolithoautotrophs living on the surface and subsurface of the chimneys.

In conclusion, stable carbon isotope ratios are very useful and may give indication of the origin of organic compounds (both gas and semivolatiles) in hydrothermal contexts, but they should be complemented with other approaches. To cite a few examples in the literature: position-specific methods have been shown to be efficient in determining the origin of deep gases (Corso and Brenna, 1997); combined analyses with noble gases (Sherwood Lollar and Ballentine, 2009); clumped isotopes is a rather new tool that would be worth trying; thermodynamic calculations could help discrimination (Reeves *et al.*, 2014); the use of radiogenic carbon has proven to be efficient in determining the source of methane (Proskurowski *et al.*, 2008); the thermal degradation experiments of biomass carried out by Konn *et al.* (2011) brought additional lines of evidence toward a plausible abiogenic origin of a portion of n-alkanes versus a likely biogenic origin of a portion of aromatic hydrocarbons and n-carboxylic acids (all >C_8_) detected in fluids from ultramafic-hosted systems.

## 6.5. Implications for the origin of life

Life may have appeared on Earth in the earliest Archean or even before in the Hadean (Russell and Hall, 1997; Rosing, 1999; Korenaga, 2013). Hydrothermal activity is relevant to Hadean and Archean Earth, as it began as soon as water condensed to form oceans, and some kind of plate tectonics (corresponding to crust formation) appeared 4.4 billion years ago (Wilde *et al.*, 2001). Also, hydrothermal systems as well as ultramafic rocks were much more abundant on primitive Earth than today (Russell *et al.*, 1988).

Although the composition, oxidation state, temperature, and pressure of the early atmosphere after the bombardment is unknown (*e.g.*, Marshall, 1994; Schoonen *et al.*, 1999), a proposed composition on which most of the scientific community agrees is domination by CO_2_ in a dense state, N_2_ and H_2_O; little amount of H_2_S, HCl, SO_2_, and elemental sulfur S^0^; and minor amounts of H_2_ and Ar (Chen and Chen, 2005; Russell and Arndt, 2005). Different lines of evidence indicate the presence of significant levels of CH_4_ (100–1000 ppm) in the atmosphere in the Archean (Pavlov *et al.*, 2000; Kasting, 2005). Fiebig *et al.* (2007) proposed an abiogenic origin of this CH_4_. Magnesium (Mg) as well as transition metals such as iron (Fe) and nickel (Ni) must have been abundant in the early ocean (Mloszewska *et al.*, 2012). Mg^2+^ together with Ca^2+^ would have been the prevalent divalent cation, while the prevalent monovalent cation was Na^+^ (Pontes-Buarque *et al.*, 2000). The ocean is considered to have been fairly acidic with a pH ∼5–6 (Russell and Arndt, 2005). Finally, almost uncontested to date, is the view that both atmosphere and ocean would have remained anoxic (oxygen-free) until the great oxidizing event postulated at 2.4 billion years ago. However, several controversial lines of evidence, including the sulfur isotopic composition of pyrites and the elemental compositions of ancient soil horizons, have been put forth to instead support the presence of appreciable amounts, or at least whiffs, of oceanic and atmospheric oxygen long before (Anbar *et al.*, 2007; Konhauser, 2009). Moreover, a recent paper by Hoashi *et al.* (2009) reports on the observation of hematite crystals in marine sediments of 3.46 Ga, which indicates that free oxygen would have existed at least locally in the oceans at that time.

As a conclusion, conditions at modern seafloor hydrothermal systems seem to be similar, to some extent, to early Earth's conditions and thus can be considered a place of primary focus in the search for the origin of life. Moreover, hydrothermal vents constitute very favorable environments for the start of life, as much in terms of protection against the sterilizing effect of giant impacts as in terms of scale. Microenvironments such as mineral surfaces favor adsorption, concentration of organics, and subsequent reactions. In addition, a hydrothermal mound provides some kind of protection (niches), physicochemical gradients, and nonequilibrium conditions that are required for the majority of macromolecules typical of the cell organization to persist as well as for the emergence of a living organism (Russell and Hall, 1997; Kompanichenko, 2009). The serpentinization process is emerging as an increasingly likely source of the energy essential for life to have emerged from CO_2_, rocks, and water on early Earth (Russell *et al.*, 1989, 2010; McCollom and Seewald, 2013). Alkaline (high pH) hydrothermal systems are thought to be even more relevant to Archean hydrothermal vents, and the Lost City hydrothermal field could provide particular insights into past mantle geochemistry and present a better understanding of the chemical constraints that existed during the evolutionary transition from geochemical to biochemical processes. In parallel and in quest of the origin of life, experimental work simulating alkaline hydrothermal vents and/or Hadean conditions is being done (Herschy *et al.*, 2014; Yamaguchi *et al.*, 2014).

## Abbreviations Used

FTT: Fischer-Tropsch-type
MAR: Mid-Atlantic Ridge
MOR: Mid-Ocean Ridge
